# Fractionated plasma separation and adsorption integrated with continuous veno-venous hemofiltration in patients with acute bipyridine herbicide poisoning

**DOI:** 10.1080/0886022X.2024.2374013

**Published:** 2024-07-05

**Authors:** Jian-Hua Dong, Minghong Zhang, Xi Yang, Bian Wu, Li Huang, Chuan Li, Yongchun Ge

**Affiliations:** National Clinical Research Center of Kidney Diseases, JinLing Hospital, Nanjing University School of Medicine, Nanjing, Jiangsu, China

**Keywords:** Poisoning, paraquat, diquat, blood purification, cytokines

## Abstract

**Objective:**

To evaluate the clinical efficacy and safety of fractionated plasma separation and adsorption combined with continuous veno-venous hemofiltration (FPSA-CVVH) treatment in patients with acute bipyridine herbicide poisoning.

**Methods:**

A retrospective analysis of 18 patients with acute bipyridine herbicide poisoning was conducted, of which 9 patients were poisoned by diquat and 9 patients by paraquat. All patients underwent FPSA-CVVH treatment. The serum cytokine levels in pesticide-poisoned patients were assessed. The efficacy of FPSA-CVVH in eliminating cytokines, the 90-d survival rate of poisoned patients, and adverse reactions to the treatment were observed.

**Results:**

Fourteen patients (77.8%) had acute kidney injuries and 10 (55.6%) had acute liver injuries. The serum cytokine levels of high mobility group protein B-1 (HMGB-1), interleukin-6 (IL-6), IL-8, interferon-inducible protein-10 (IP-10), monocyte chemotactic protein-1 (MCP-1), and macrophage inflammatory protein-1β (MIP-1β) were significantly elevated. A total of 41 FPSA-CVVH treatment sessions were administered. After a single 8-h FPSA-CVVH treatment, the decreases in HMGB-1, IL-6, IL-8, IP-10, MCP-1, and MIP-1β were 66.0%, 63.5%, 73.3%, 63.7%, 53.9%, and 54.1%, respectively. During FPSA-CVVH treatment, one patient required a filter change due to coagulation in the plasma component separator, and one experienced a bleeding adverse reaction. The 90-d patient survival rate was 50%, with 4 patients with diquat poisoning and 5 patients with paraquat poisoning, and both liver and kidney functions were restored to normal.

**Conclusion:**

Cytokine storms may play a significant role in the progression of multiorgan dysfunction in patients with acute bipyridine herbicide poisoning. FPSA-CVVH can effectively reduce cytokine levels, increase the survival rate of patients with acute bipyridine herbicide poisoning, and decrease the incidence of adverse events.

## Introduction

Acute pesticide poisoning often leads to organ dysfunction and structural damage, causing disease and even death. Diquat and paraquat are nonselective and quick-acting killing herbicides that are bipyridine compounds. According to the World Health Organization’s classification of pesticides, both diquat and paraquat are considered moderately toxic pesticides, with paraquat posing a fatal risk [1]. Although safely used in agriculture, diquat and paraquat intoxication are associated with an estimated 50–70% and 20–60% mortality, respectively [2,3]. Acute bipyridine herbicide poisoning can cause serious effects, such as lung, kidney, liver, and heart failure, leading directly to death. The toxicological mechanisms of diquat and paraquat poisoning, which may cause tissue and organ damage through direct cytotoxic effects, oxidative stress responses, immune-inflammatory responses, and other pathways, are not yet fully understood [4,5]. Notably, there are no specific antidotes or conclusive effective treatments for either.

Both diquat and paraquat are small molecule, water soluble toxins with molecular weights of 184.24 and 186.25 Da, respectively, but their protein binding rates are unknown. Hemopurification technology, an essential treatment method for poisoning, can effectively reduce blood toxin levels and mitigate organ damage. Hemodialysis, hemoperfusion (HP), and continuous veno-venous hemofiltration (CVVH) have been used to treat acute bipyridine herbicide poisoning [6–10]. However, the Extracorporeal Treatments in Poisoning Workgroup (EXTRIP) has not yet released recommendations regarding hemopurification treatment for diquat and paraquat poisoning. In 2017, we developed the technique of fractionated plasma separation and adsorption (FPSA)-integrated with CVVH for the treatment of severely poisoned patients [11]. In this study, the serum cytokine levels in patients with acute bipyridine herbicide poisoning are retrospectively observed, and the effectiveness and safety of FPSA-CVVH are evaluated in the context of diquat and paraquat poisoning, providing a clinical basis for the hemopurification treatment of bipyridine herbicide poisoning.

## Materials and methods

### Patient selection

A total of 42 patients with acute bipyridine herbicide poisoning were hospitalized at Jinling Hospital between January 2017 and December 2022.

The inclusion criteria were as follows: (1) pesticide dosage reached or exceeded the toxic lethal dose; (2) pesticide poisoning grade was moderate to severe or explosive; (3) organ dysfunction, such as coma, acute kidney injury (AKI), acute liver injury (ALI), requiring mechanical ventilation or vasoactive drug therapy; and (4) FPSA-CVVH treatment.

**Figure 1.**
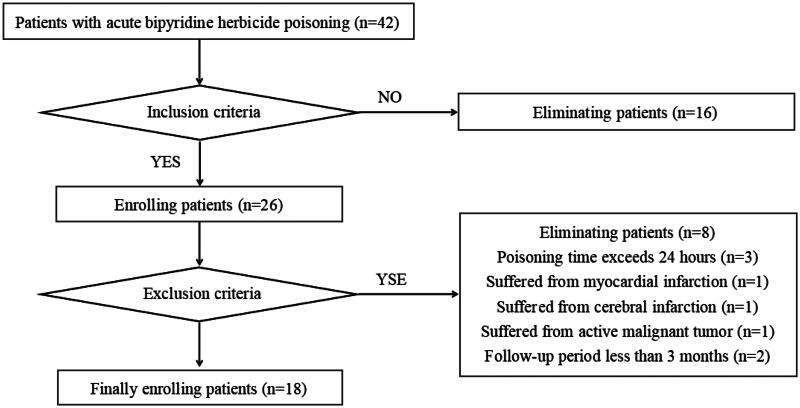
Flowchart of the process of enrolling patients with acute bipyridine herbicide poisoning treated with FPSA-CVVH.

The exclusion criteria were as follows: (1) poisoning time exceeding 24 h; (2) myocardial infarction, cerebral infarction, intracranial hemorrhage, or severe liver disease (glutamic pyruvic transaminase [GPT] ≥ 3 times the normal upper limit), chronic renal insufficiency (estimated glomerular filtration rate [eGFR] < 60 mL/min), or active malignant tumor within one month before poisoning; and (3) a follow-up period of less than 3 months. Eighteen patients were ultimately included in this study (Figure 1).

This study was reviewed by the Ethics Committee of Jinling Hospital (2022DZKY-078-02).

### Related definitions

The related definitions for diquat poisoning classification are as follows [12], classified by the dose of diquat (20% diquat dibromide) ingested: (1) mild poisoning, intake dose <9.35 mL; (2) moderate to severe poisoning, intake dose 9.35–112.20 mL; and (3) explosive poisoning, intake dose >112.2 mL. The lethal dose of diquat is 6–12 g (equivalent to 30–60 mL of 20% diquat dibromide) [13].

Paraquat poisoning was classified as follows [14]: (1) mild poisoning, with an intake dose <20 mg/kg; (2) moderate to severe poisoning, with an intake dose of 20–40 mg/kg; and (3) explosive poisoning, with an intake dose >40 mg/kg. The lethal dose of paraquat is 20–40 mg/kg (equivalent to 5–15 mL of 20% paraquat aqueous solution) [15].

AKI was diagnosed based on the guidelines of Kidney Disease: Improving Global Outcomes (KDIGO) [16]^,^ and the eGFR was calculated by the CKD-EPI formula. Acute lung injury (ALI) was diagnosed based on the criteria of serum GPT > 70 U/L and/or serum total bilirubin >34.2 μmol/L.

### FPSA-CVVH treatment procedure

**Figure 2.**
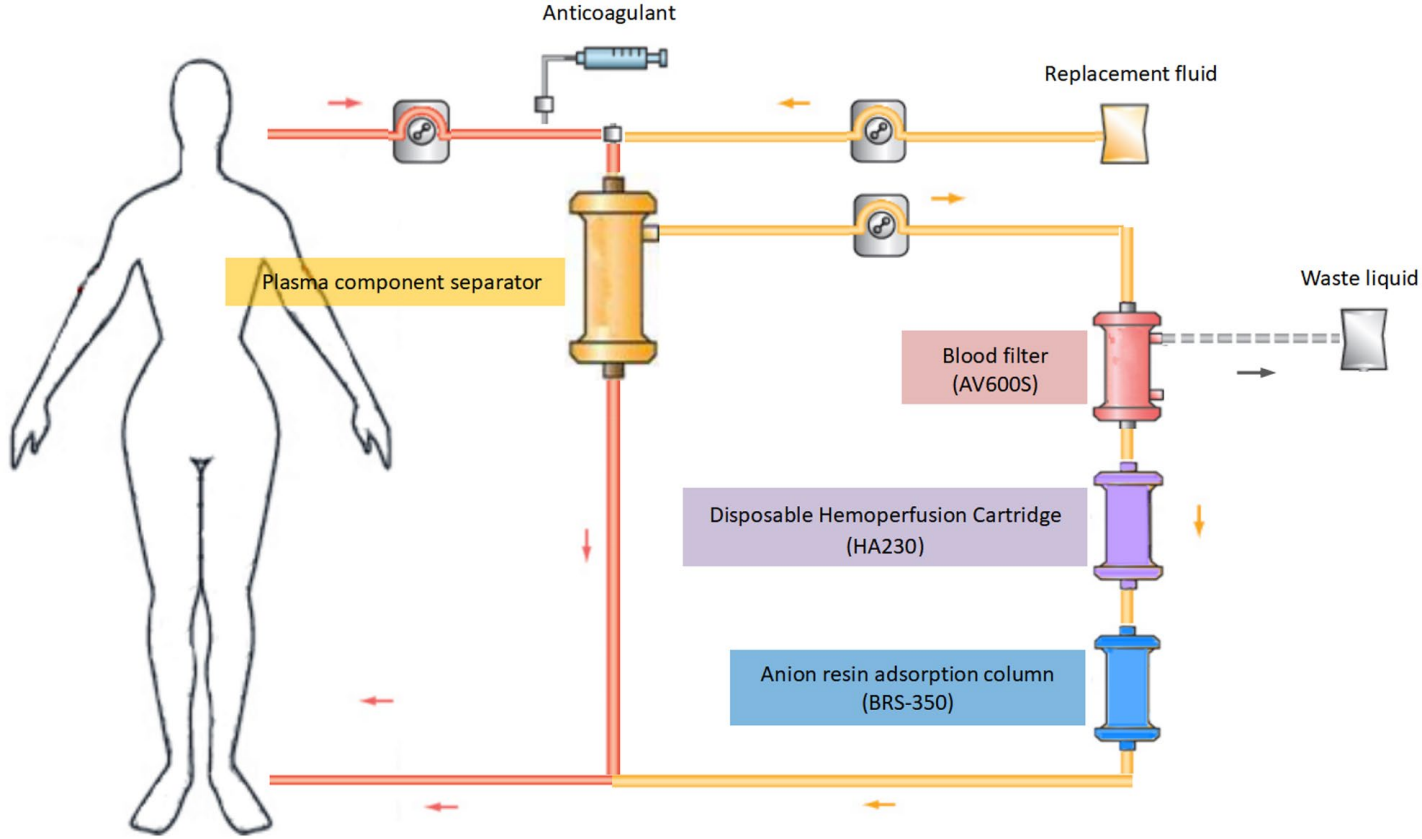
Schematic diagram of FPSA-CVVH treatments. Whole blood was separated by a plasma component separator (EC40W), with the blood flow rate and the plasma separation rate set at 220–250 and 90–100 mL/min, respectively. The separated plasma was filtered through a blood filter (AV600) at an ultrafiltration rate of 66.7 mL/min. Then, the concentrated plasma was added to a disposable hemoperfusion cartridge (HA230) and an anion resin adsorption column (BRS-350) and returned to the body. Bicarbonate replacement fluid at a speed of 4000 mL/h in predilution mode was added synchronously before EC40W.

A double-lumen indwelling central venous catheter was used for vascular access. The anticoagulant was regional citrate combined with low-dose, low-molecular weight heparin. A schematic diagram of extracorporeal circulation is shown in Figure 2. Whole blood was collected through a membrane-type plasma component separator (EC40W, Asahi Kasei Medical Co., Ltd., Tokyo, Japan). The blood flow rate was 220–250 mL/min, and the plasma separation rate was 90–100 mL/min. The plasma was filtered through a blood filter (AV600S, Fresenius Medical Care AG & Co., Bad Homburg, Germany) at an ultrafiltration rate of 66.7 mL/min, and the plasma was adsorbed by a disposable HP cartridge (HA230, Jafron Biomedical Co., Ltd., Zhuhai, China) and a selective plasma component adsorber (BRS-350, Asahi Kasei Medical Co., Ltd., Japan) and then returned back into the body. At the same time, the bicarbonate replacement solution was injected before the EC40W separator at a rate of 66.7 mL/min, and the treatment time of one FPSA-CVVH session was 8 h. Continuous CVVH treatment was continued for 16 h and continuous treatment lasted for 48 h. Then, the doctor adjusted the treatment duration based on the improvement of the patient’s poisoning symptoms.

### Clinical evaluation indices

Patient age, sex, underlying disease (hypertension, diabetes, liver and kidney diseases, etc.), vital signs, acute physiological function, and chronic health status II (APACHE II) score, poisoning severity score (PSS) [17], arterial blood gas analysis, complete blood count, coagulation function, liver and kidney function, electrolytes, and the myocardial enzyme spectrum were retrieved from the medical records information system. Pesticide poisoning time, pesticide name, intake method and dose, clinical symptoms of poisoning, and treatment were also recorded.

The sodium bicarbonate/dithionite test was used to detect the urine of patients with bipyridine pesticide poisoning [4,14]. Blood and urine pesticide concentrations were tested using a liquid chromatography–mass spectrometry instrument. Our hospital cannot determine the pesticide concentration in blood and urine, and only some patients send samples to the poison identification center to complete pesticide concentration monitoring. Serum cytokines were detected using the enzyme-linked immunosorbent assay method.

Changes in clinical manifestations and serum cytokine concentrations after poisoning were recorded. Changes in vital signs and clinical indicators before and after FPSA-CVVH treatment were evaluated. The rate of decrease in serum cytokine levels in patients after 8 and 24 h of FPSA-CVVH treatment was assessed, as was the rate of change in serum cytokine levels after 24 h of treatment. Coagulation in the filter during treatment and complications such as bleeding in the patients were documented. In-hospital mortality, 90-d mortality rate, and the occurrence and degree of chronic organ damage were recorded.

$$\begin{array}{l} \text{Cytokine decline rate}\\ =\;\left(\begin{array}{l} \text{pretreatment concentration }\\ -\text{ 8 h or 24 h posttreatment concentration} \end{array}\right)\\ \;/\text{pretreatment concentration }\times \;100\% \end{array}$$

$$\begin{array}{l} \text{Cytokine rebound rate }\\ =\;\left(\begin{array}{l} \text{24 h posttreatment concentration }\\ -\text{ 8 h posttreatment concentration} \end{array}\right)\\ \;/\text{8 h posttreatment concentration }\times \text{ 100}\% \end{array}$$

### Statistical analysis

All the data were statistically analyzed and processed using IBM SPSS version 20 software (IBM SPSS Inc., Chicago, IL). Quantitative data are expressed as the mean ± standard deviation or median and interquartile range. Serum cytokine levels before and after FPSA-CVVH and at 24 h were compared using repeated-measures analysis of variance (normal distribution; if the spherical hypothesis *p* < 0.01; Greenhouse–Geisser correction was needed) or the Friedman test (nonnormal distribution). The Bonferroni *post hoc* correction was used for intergroup comparisons. Changes in the clinical and laboratory indicators before and after treatment were analyzed using paired sample *t*-tests (normal distribution) or Wilcoxon tests (nonnormal distribution). Count data are expressed as percentages, and the Chi-square test was used for intergroup comparisons. *p* < 0.05 was considered to indicate statistical significance.

## Results

### Clinical characteristics

Eighteen patients with acute bipyridine herbicide poisoning, including 7 males and 11 females, who had a median age of 31 (20, 50) years, received FPSA-CVVH treatment. One patient had hypertension, and 2 patients had diabetes. Six patients (33.3%) received mechanical ventilation, and 5 patients (27.8%) required vasopressor drugs. Fourteen patients (77.8%) had AKI, and 10 patients (55.6%) had ALI.

Nine patients were poisoned by paraquat, and 9 were poisoned by diquat, all due to intentional ingestion. All patients had a PSS score of 3, indicating severe, life-threatening symptoms or signs of poisoning and underwent emetic induction, gastric lavage or catharsis, and supportive treatment. Patients with stable circulation were given diuretics to promote pesticide excretion. Patient clinical details are shown in Table 1.

**Table 1.** The clinical characteristics of the patients with acute bipyridine herbicide poisoning at baseline.

	Diquat poisoning (*n* = 9)	Paraquat poisoning (*n* = 9)	*p* Value
Age (years)	28.0 (22.5,53.0)	32.0 (16.0,50.5)	0.860
Male/female	2/7	5/4	1.000
APACHE II (score)	4.0 (1.5,13.5)	3.0 (2.0,5.5)	0.533
White blood cell (×10^9^/L)	18.3 (10.94,25.25)	9.9 (8.79.0,1.57)	0.102
Hemoglobin (g/L)	143.0 ± 12.9	132.0 ± 16.1	0.131
Platelet count (×10^9^/L)	241.1 ± 93.4	242.0 ± 106.1	0.985
Blood urea nitrogen (mmol/L)	5.8 (4.2,8.9)	7.1 (5.5,9.2)	0.377
Serum creatinine (μmol/L)	118.4 (62.2,224.8)	157.0 (114.0,187.5)	0.691
GPT (U/L)	82.0 (25.0,488.0)	64.0 (28.5,149.0)	0.401
Total bilirubin (μmol/L)	16.9 (14.8,24.1)	16.5 (11.9,38.9)	0.757
Creatine kinase (U/L)	161.0 (83.0,514.3)	155.0 (41.5,423.0)	0.630
Serum cytokine (pg/mL)			
HMGB-1	84827 (1758.0,22421.2)	13442.5 (5585.0,20491.7)	0.480
IL-1β	6.30 (4.45,61.02)	7.87 (4.48,26.48)	0.742
IL-6	364.6 (153.0,2195.5)	664.9 (96.0,1851.0)	0.828
IL-8	546.0 (174.2,4330.8)	270.8 (118.6,1512.5)	0.386
IL-10	12.56 (7.01,111.18)	11.48 (6.21,56.39)	0.914
TNF-α	9.10 (5.52,16.43)	12.42 (9.62,37.02)	0.212
IP-10	41.33 (15.59,83.30)	45.83 (26.74,91.34)	0.664
MCP-1	811.9 (705.4,2100.5)	1387.6 (22.7,3126.0)	1.000
MIP-1β	438.3 (235.5,1191.9)	271.9 (239.8,726.3)	0.448
Acute renal injury, *n*(%)	6 (66.7)	8 (88.9)	0.576
Acute liver injury, *n*(%)	5 (55.6)	5 (55.6)	1.000
Mechanical ventilation, *n*(%)	4 (44.4)	2 (22.2)	0.620
Vasopressor drug, *n*(%)	4 (44.4)	1 (11.1)	0.294

GPT: glutamic pyruvic transaminase; HMGB-1: human high mobility group protein B1; IL-1β: Interleukin 1β; TNF-α: tumor necrosis factor α; IP-10: interferon inducible protein 10; MCP-1: monocyte chemoattractant protein 1; MIP-1β: macrophage inflammatory protein 1β

### Diquat poisoning

The ingested pesticide was a 20% diquat aqueous solution, and the active ingredient was diquat dibromide. The median ingested dose was 120 (28, 175) mL, with five patients exceeding 112.2 mL (i.e., explosive poisoning). The primary clinical manifestations included oral ulcers, nausea, vomiting, abdominal pain, diarrhea, and other gastrointestinal symptoms. Six patients had AKI, five patients had ALI, and four patients required mechanical ventilation and vasoactive drugs. The blood concentration of diquat dibromide was 165.0 (140.5, 3458.0) ng/mL, and the urine concentration of diquat dibromide was 6874.0 (1869.9, 8206.5) ng/mL in six patients.

### Paraquat poisoning

The ingested pesticide was a 20% paraquat aqueous solution. Two patients mixed paraquat with alcohol before ingestion. The median ingested amount was 40 (20,55) mL or 101.7 (65.5,198.0) mg/kg, exceeding lethal doses. Clinical presentations included pain in the mouth, throat, behind the sternum, and abdomen, accompanied by nausea and vomiting, and one patient developed a gastrointestinal perforation. Six patients reported chest tightness and shortness of breath, two rapidly developed acute respiratory distress syndrome, and one had subcutaneous and mediastinal emphysema. Eight patients developed AKI and five patients had liver function impairment. Two patients showed symptoms of neurotoxicity, such as headaches, restlessness, convulsions, and altered consciousness. Two patients reported palpitations, 1 of whom experienced circulatory failure requiring vasoactive drugs. The blood concentrations of paraquat in three patients were 96, 86.5, and 286 ng/mL, while the urine concentrations of paraquat were 2085, 2524, and 317 ng/mL, respectively. All patients were treated with methylprednisolone (500 mg/d for 3 d, reduced to 40 mg/d thereafter) and cyclophosphamide (15 mg/[kg·d]) for 2 d.

### Serum cytokines after poisoning

Serum cytokines, including high mobility group protein B-1 (HMGB-1), interleukin-6 (IL-6), IL-8, interferon inducible protein-10 (IP-10), monocyte chemotactic protein-1(MCP-1), and macrophage inflammatory protein-1β (MIP-1β), were significantly elevated, with median concentrations of 11225.5, 550.13, 284.51, 43.01, 811.94, and 293.18 pg/mL, respectively. However, IL-1β, IL-10, and tumor necrosis factor-α (TNF-α) showed less prominent increases, with median concentrations of 6.3, 12.01, and 10.99 pg/mL, respectively. There was no difference in serum cytokine levels between diquat-poisoning patients and paraquat-poisoning patients (Table 1).

### Clinical efficacy of FPSA-CVVH treatments

The median interval between poisoning and FPSA-CVVH treatment was 5.5 (3.0, 8.8) h. A total of 41 FPSA-CVVH sessions were administered to the 18 patients, and each patient underwent 2–3 sessions. Vascular access was achieved *via* a femoral venous catheter. The mean treatment blood flow rate was 240.3 ± 14.1 mL/min, and the mean plasma separation rate was 90.8 ± 4.0 mL/min (with 66.7 mL/min of replacement fluid).

Following FPSA-CVVH treatment, white blood cell counts, C-reactive protein levels, blood urea nitrogen levels, serum creatinine levels, GPT levels, total bilirubin levels, and creatine kinase levels decreased significantly (Table 2).

**Table 2.** The clinical characteristics of the patients with acute bipyridine herbicide poisoning before and after FPSA-CVVH treatment.

	Before FPSA-CVVH treatment (*n* = 18)	After FPSA-CVVH treatment (*n* = 18)	*p* Value
White blood cell (×10^9^/L)	12.04 (9.09,21.72)	9.19 (6.45,12.2)	0.004
Hemoglobin (g/L)	137.5 ± 15.26	133.0 ± 15.4	0.151
Platelet count (×10^9^/L)	241.6 ± 97.0	219.9 ± 92.2	0.078
aPTT (s)	26.19 ± 12.23	31.11 ± 8.61	0.198
PT (s)	11.91 ± 4.08	13.84 ± 2.35	0.158
C-reactive protein (mg/L)	8.7 (4.95,64.73)	1.15 (0.63,5.48)	0.001
Blood urea nitrogen (mmol/L)	5.85 (5.38,8.43)	3.25 (2.45,5.98)	0.001
Serum creatinine (μmol/L)	153.6 (81.0,187.8)	59.5 (48.9,94.6)	<0.001
GPT (U/L)	65.5 (25.0,195.3)	23.0 (16.5,48.5)	<0.001
Total bilirubin (μmol/L)	16.7 (13.7,23.5)	9.9 (7.6,15.8)	0.001
Creatine kinase (U/L)	155.0 (66.5,423.0)	61.5 (38.0,159.5)	<0.001
Sodium (mmol/L)	138.11 ± 3.75	138.09 ± 2.16	0.254
Potassium (mmol/L)	3.72 ± 0.37	4.10 ± 0.25	0.554
Calcium (mmol/L)	2.31 ± 0.25	2.21 ± 0.15	0.414
Total carbon dioxide (mmol/L)	20.31 ± 3.86	23.28 ± 2.62	0.002
pH	7.37 ± 0.11	7.40 ± 0.04	0.210
PO_2_ (mmHg)	88.0 (76.0,101.5)	98.5 (78.8,113.5)	0.683
PCO_2_ (mmHg)	33.3 ± 6.9	35.3 ± 4.1	0.124

aPTT: activated partial thromboplastin time; PT: prothrombin time; GPT: glutamic pyruvic transaminase; PO_2_: oxygen partial pressure; PCO_2_: partial pressure of carbon dioxide

The levels of the cytokines HMGB-1, IL-6, IL-8, IP-10, MCP-1, and MIP-1β were significantly decreased after single FPSA-CVVH treatment for 8 h, and the median reduction rates were 66.0%, 63.5%, 73.3%, 63.7%, 53.9%, and 54.1%, respectively. After 16 h of sequential CVVH treatment, the cytokine levels rebounded compared with those after 8 h of treatment, but they were still lower than those before treatment (Table 3).

**Table 3.** Serum cytokine clearance at 8 and 24 h of treatment with a single FPSA-CVVH session (18 patients, 41 FPSA-CVVH sessions).

Cytokine (pg/mL)	Pretreatment	8 h posttreatment	24 h posttreatment	8 h decline rate (%)	24 h decline rate (%)	24 h rebound rate (%)
HMGB-1	9541.0(3075.0,19421.1)	3390.0(1733.0,5919.8)	6198.5(2544.3,10111.6)	66.0(50.6,75.2)	34.8(18.3,50.5)	63.5 (26.3, 129.2)
IL-1β	5.7(4.5,24.2)	2.9(2.4,4.5)	4.5(3.4,9.6)	48.5(44.7,80.2)	27.6(19.8,55.9)	62.9 (40.3,89.8)
IL-6	635.0(53.7,1795.0)	168.2(26.9,505.2)	433.1(51.6,717.6)	63.5(53.9,79.7)	43.4(26.6,54.7)	36.4 (22.8, 62.5)
IL-8	199.1(125.1,1398.0)	49.9(31.2,150.0)	129.9(83.7,749.9)	73.3(57.4,90.6)	29.4(17.5,63.8)	195.8 (89.6, 275.7)
IL-10	11.8(7.1,23.3)	5.0(3.6,8.4)	8.6(6.4,13.1)	54.5(42.1,62.7)	31.9(16.3,62.5)	72.8 (36.8,112.7)
TNF-α	10.2(7.9,13.4)	6.0(4.3,8.0)	8.95(7.0,11.5)	43.0(34.4,59.0)	30.2(6.7,61.7)	47.9 (22.1, 75.9)
IP-10	45.9(27.6,81.2)	12.3(9.6,32.4)	34.8(27.4,48.0)	63.7(53.1,72.7)	28.9(11.4,49.2)	150.1 (108.9, 243.7)
MCP-1	780.2(204.2,2432.0)	360.9(86.1,682.0)	533.0(153.7,1260.0)	53.9(47.7,66.2)	16.3(9.7,33.0)	72.8 (60.3, 97.4)
MIP-1β	289.1(165.9,469.1)	167.44(107.3,197.3)	231.4(165.9,469.1)	54.1(41.8,69.9)	33.9(30.6,48.8)	63.7 (37.3, 121.9)

GPT: glutamic pyruvic transaminase; HMGB-1: human high mobility group protein B1; IL-1β: Interleukin 1β; TNF-α: tumor necrosis factor α; IP-10: interferon inducible protein 10; MCP-1: monocyte chemoattractant protein 1; MIP-1β: macrophage inflammatory protein 1β

The mean arterial pressure (95.8 ± 14.5 mmHg *vs.* 93.6 ± 11.5 mmHg, *p* = 0.445) and heart rate (86.0 ± 11.2 beats/min *vs.* 88.5 ± 12.3 beats/min, *p* = 0.436) before and after FPSA-CVVH treatment, respectively, remained stable. No new vasopressor drugs or increased doses were administered during treatment.

Severe coagulation in the EC40W plasma component separator was observed in one patient, although treatment was continued after the filter was replaced. Another patient exhibited adverse bleeding, which was evident as gross hematuria (observed during catheterization). The bleeding improved after the withdrawal of anticoagulants. During FPSA-CVVH treatment with basic anticoagulation using regional citrate, perioral or distal extremity numbness, tetany, hypotension, or a prolonged Q-T interval were not reported.

### Prognosis

**Figure 3.**
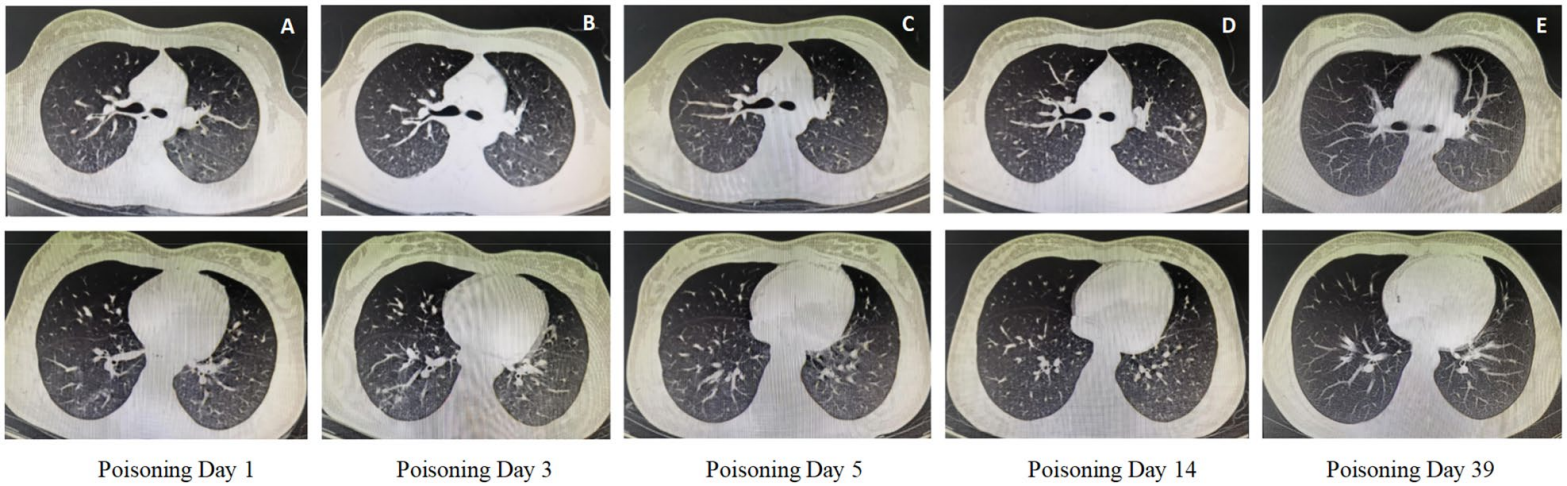
Changes in chest CT findings in patients with paraquat poisoning during FPSA-CVVH treatment. (A) The right lung and the lower lobe of the left lung were scattered in spots and small patches of ground glass density shadows, and the boundary was unclear; (B) The lung inflammatory shadow progressed compared with that on the 1st day; (C) The lung inflammatory shadow was slightly absorbed compared with that on the third day; (D) The lungs were scattered with little inflammatory shadow; (E) No abnormalities were found on chest CT.

The median length of hospital stay for patients with acute bipyridine herbicide poisoning was 4.5 (30,11.0) d. For survivors of paraquat poisoning, lung imaging showed only minimal inflammation in the early stages of the disease, and dynamic monitoring did not show progression (Figure 3). Liver and kidney functions were normalized in patients with AKI and ALI during the follow-up period.

The 90-d mortality rate of the patients was 50.0%. Nine patients died during the follow-up period, including four patients with diquat poisoning and five patients with paraquat poisoning. Among them, six patients died due to multiple organ failure during hospitalization, while three patients died due to pulmonary fibrosis and respiratory failure caused by paraquat after abandoning treatment. The baseline serum HMGB-1, IL-1β, IL-6, and IL-8 levels were significantly greater in deceased patients than in surviving patients (Table 4).

**Table 4.** Comparison of the serum cytokines between surviving and deceased patients in acute bipyridine herbicide poisoning.

Serum cytokine (pg/mL)	Surviving patients (*n* = 9)	Deceased patients (*n* = 9)	*p* Value
HMGB-1	4925.0 (2291.8, 8526.9)	19658.7 (14285.1, 28535.7)	0.001
IL-1β	4.48 (4.48, 6.62)	17.74 (6.77,137.09)	0.009
IL-6	125.22 (26.26, 525.16)	1689.5 (522.66,1914.00)	0.016
IL-8	155.79 (105.59, 707.70)	1428.02 (248.11, 3709.25)	0.027
IL-10	11.3 (8.02, 13.54)	17.79 (4.79, 145.2)	0.674
TNF-α	10.02 (6.90, 11.21)	20.71 (9.35, 39.17)	0.066
IP-10	32.34 (26.25, 56.23)	79.66 (18.08, 125.18)	0.248
MCP-1	805.44 (238.96, 3281.25)	1397.03(157.56,2396.75)	0.674
MIP-1β	271.98 (244.39,406.40)	381.20 (242.19,2089.25)	0.401

HMGB-1: human high mobility group protein B1; IL-1β: Interleukin 1β; TNF-α: tumor necrosis factor α; IP-10: interferon inducible protein 10; MCP-1: monocyte chemoattractant protein 1; MIP-1β: macrophage inflammatory protein 1β

## Discussion

This study investigated the clinical manifestations of patients with acute bipyridine herbicide poisoning, as well as the prognosis and adverse events of patients after FPSA-CVVH treatment. The results revealed that patients with bipyridine herbicide poisoning had a significant increase in cytokine levels, especially in nonsurvivors, which may be one of the potential pathogenic mechanisms leading to organ injury. FPSA-CVVH can effectively reduce cytokine levels and increase patient survival rates.

During the early stages of diquat and paraquat poisoning, the levels of cytokines such as HMGB-1, IL-6, IL-8, IP-10, MCP-1, and MIP-1β were significantly elevated. Thus, in addition to the direct organ damage caused by pesticides, the release of cytokines might play a key role in the progression of pesticide poisoning. Diquat and paraquat primarily cause intrinsic cellular injury through oxidative stress responses. They also lead to excessive activation of immune cells and a cytokine storm, impacting the kidneys, liver, lungs, digestive tract, and nervous system, which can result in life-threatening multiorgan dysfunction [4,18]. Currently, there are no specific antidotes for diquat or paraquat poisoning. Given that the prognosis of poisoned patients is significantly related to the dose of the poison, early measures to remove toxins and cytokines are critical for successful treatment.

Blood purification can effectively reduce blood toxin levels and alleviate organ damage, making blood purification a vital treatment for poisoning [19]. Due to the diversity of pesticides, individual dosage variations, and distinct toxicological characteristics, randomized controlled clinical trials on blood purification treatments for pesticide poisoning are rare. Bipyridine compounds have a large distribution volume, and after being absorbed into the bloodstream, they quickly distribute to various tissues and organs, with only a small fraction present in the blood [4,20,21]. Although blood purification treatment can remove some toxins, it is challenging to significantly reduce the toxic load in organs.

Presently, the latest Chinese expert consensus on paraquat poisoning (2022 version) explicitly recommends using HP combined with CVVH or hemodialysis [2]. However, concerning diquat poisoning, due to the lack of substantial clinical evidence, the latest Chinese expert consensus (2020 version) suggests performing continuous renal replacement therapy in patients with AKI [3]. Blood perfusion followed by hemodialysis or hemodiafiltration might improve the condition and prognosis of patients with severe poisoning. Early HP followed by CVVH treatment in patients with paraquat poisoning can quickly reduce the blood paraquat concentration, alleviate organ function damage, and improve patient survival rates [6,7]. However, an uncontrolled study conducted by Koo et al. [22] showed that the number of survival days was prolonged, but there was no difference in mortality when HP was given along with CVVH compared to HP alone in a group of 80 patients with paraquat poisoning, and mortality between these two groups was found to be 63.6% *vs.* 66.7%^.^ When dealing with pesticide poisonings whose toxicological properties are not yet clear, mixed pesticide poisonings, or poisonings with doses exceeding lethal levels, single or sequential blood purification modalities may not effectively and rapidly reduce the body’s toxin burden and cytokine levels. Using combined blood purification techniques can aid in the removal of toxins with different toxicological properties, increase cytokine clearance, and support essential organ functions.

The novel blood purification method FPSA-CVVH, constructed by our center, is used for the treatment of severely poisoned patients. This mode employs a plasma component separator to separate the plasma. The plasma first passes through a filter to clear water-soluble toxins and then through two adsorption columns (HA230 and BRS350) to remove lipid-soluble toxins, macro- to middle-molecule toxins, and protein-bound toxins. This avoids direct contact of blood cells with the filter and adsorption columns, reduces the dose of anticoagulants, lowers the risk of filter clotting, prevents nonprotein components from contacting the adsorption column, reduces the saturation speed of the column, improves toxin removal efficiency, simultaneously removes cytokines, adjusts water, acid–base and electrolyte balance, and achieves liver and kidney function support treatment. The results of this study showed that after 8 h of FPSA-CVVH treatment, the levels of the cytokines HMGB-1, IL-6, IL-8, IP-10, MCP-1, and MIP-1β decreased significantly, with reduction rates all exceeding 50%. After FPSA-CVVH treatment, poisoned patients did not develop new cases of AKI or ALI, and lung damage did not progress in surviving paraquat-poisoned patients. However, during sequential CVVH, the cytokine clearance effect decreased compared to that of FPSA-CVVH treatment, and the cytokine levels rebounded. These results indicated that in the early stage of acute poisoning, cytokine storms continue to play a role. After stopping FPSA-CVVH, CVVH alone cannot efficiently clear medium- and large-molecule cytokines. Longer treatment with FPSA-CVVH may be beneficial for improving the success rate of treatment. In addition, FPSA-CVVH treatment is characterized by good tolerance and no serious adverse reactions. The patient’s mean arterial pressure and heart rate remained stable during treatment. The incidence of adverse reactions such as bleeding and filter coagulation was low, at only 4.9% (2/41).

Patients with moderate-to-severe acute bipyridine herbicide poisoning, especially explosive poisoning, progress rapidly in a short time to multiple organ failure, and most of them die within 24–48 h [2,3]. Whether blood purification can improve the prognosis of patients with severe or explosive pesticide poisoning is still debated, as the current research results are limited and lack strong supporting evidence. Sabzghabaee et al. [23] and others reported that all patients who died had ingested more than 40 mg/kg paraquat and underwent hemodialysis. A multicenter retrospective study conducted by Wu et al. [24] showed that when more than 50 mL of diquat was ingested, the mortality rate of patients treated with HP ranged from 56.6% to 85.7%. FPSA-CVVH has improved the treatment success rate for patients with moderate-to-severe poisoning caused by paraquat and diquat (including explosive poisoning) to 50%.

This study is a retrospective study and has certain limitations. The sample size was small, and only some patients were monitored for diquat and paraquat concentrations in blood and urine; therefore, we could not obtain pharmacokinetic data on how FPSA-CVVH clears bipyridine pesticides. The longer the interval between poisoning time and the start of blood purification treatment, the lower the effectiveness of the blood purification treatment. Blood perfusion within 4 h of paraquat poisoning can reduce the mortality rate of poisoned patients [25]. In this study, the median time interval from poisoning to FPSA-CVVH treatment exceeded 4 h, which may have affected the effectiveness of FPSA-CVVH in treating diquat and paraquat poisoning. In the future, prospective controlled clinical studies are needed to further clarify the long-term effects of FPSA-CVVH on the prognosis of poisoned patients and its application value in other disease areas.

In summary, cytokine storms may play a significant role in the progression of multiorgan dysfunction in patients with acute bipyridine herbicide poisoning. FPSA-CVVH can effectively reduce cytokine levels, increase the success rate of treatment for acute bipyridine herbicide poisoning, and has a low incidence of adverse reactions.

## Data Availability

The data given this article are the raw data required to reproduce these findings and cannot be shared at this time as the data also forms part of an ongoing study.
